# Injectable Nanocomposite Hydrogel for Enhanced Mild Photothermal Therapy of Oral Cancer via Oxygen-Independent Thermodynamic Therapy

**DOI:** 10.3390/polym18212767

**Published:** 2026-11-06

**Authors:** Wenjuan Chen, Hui Lv, Xiaoyan Zhang, Jun Zhang

**Affiliations:** 1 Department of Central Laboratory, Tianjin Stomatological Hospital, School of Medicine, Nankai University, Tianjin 300041, China; zhangxiaoyan@nankai.edu.cn; 2 Tianjin Key Laboratory of Oral and Maxillofacial Function Reconstruction, Tianjin Stomatological Hospital, Nankai University, Tianjin 300041, China; 2120242035@mail.nankai.edu.cn; 3 Stomatology Institute of Nankai University, Tianjin 300041, China; 4 Department of Oromaxillofacial-Head and Neck Oncology, Tianjin Stomatological Hospital, School of Medicine, Nankai University, Tianjin 300041, China

**Keywords:** polypseudorotaxane, host–guest assembly, nanocomposite hydrogel, hyaluronic acid, Pluronic F127, mild photothermal therapy, oxygen-independent thermodynamic therapy, oral squamous cell carcinoma

## Abstract

Polypseudorotaxane-based polymeric networks provide a versatile framework for constructing injectable hydrogels with dynamic assembly and multifunctional therapeutic capabilities. Herein, we developed an injectable nanocomposite hydrogel (IA@PFHA) based on a polypseudorotaxane network formed through host–guest assembly of α-cyclodextrin-modified hyaluronic acid (HA-αCD) and indocyanine green (ICG)-loaded Pluronic F127 nanoparticles (ICG@PF127), with 2,2′-azobis[2-(2-imidazolin-2-yl)propane] dihydrochloride (AIPH) incorporated as an oxygen-independent radical initiator. The resulting network enabled the co-incorporation of ICG and AIPH while exhibiting shear-thinning injectability, providing a polymeric platform for localized integration of photothermal and radical-generating functions. Upon 808 nm irradiation, IA@PFHA generated mild hyperthermia below 45 °C while promoting AIPH-derived radical generation. This design integrates mild photothermal therapy (m-PTT) with oxygen-independent thermodynamic therapy (TDT), thereby addressing the limited thermal stress of mild heating and the therapeutic constraints associated with hypoxic tumor microenvironments. In vitro, IA@PFHA retained its radical-generating capability under hypoxic conditions and enhanced NIR-induced cytotoxicity against CAL 27 cells. In vivo, IA@PFHA combined with NIR irradiation achieved greater tumor growth inhibition than the AIPH-free hydrogel and free formulation, despite comparable photothermal responses among ICG-containing groups. Overall, this study demonstrates a polypseudorotaxane-based polymeric hydrogel platform that integrates complementary photothermal and oxygen-independent radical-generating functions within an injectable network, highlighting the potential of dynamic polymeric network design for localized combination therapy for OSCC.

## 1. Introduction

Oral squamous cell carcinoma (OSCC) is the most prevalent histological subtype of oral malignancies and exhibits high malignancy, aggressive invasion, and a marked tendency toward lymph node metastasis [1,2]. Surgical resection remains the primary treatment for OSCC, with radiotherapy and chemotherapy commonly incorporated into multimodal regimens to reduce the likelihood of disease recurrence and metastasis [3,4]. However, these treatments often cause some unavoidable side effects, such as tissue defects and osteonecrosis [5,6]. Therefore, more precise and effective treatment modalities are urgently needed for OSCC management [7]. Photodynamic therapy (PDT) and photothermal therapy (PTT) have gained considerable interest as potential treatment modalities for OSCC because of their high spatial precision, limited systemic toxicity, and minimally invasive characteristics [7,8,9]. Nevertheless, the efficacy of PDT is highly dependent on the oxygen concentration within tumor tissues [10,11]. Unfortunately, intratumoral hypoxia is a hallmark of OSCC driven by rapid tumor growth and abnormal tumor vessels [12], which greatly limits the generation of singlet oxygen (^1^O_2_) during PDT. While PTT can ablate tumors through photothermal conversion, achieving effective tumor ablation often requires high temperatures, which may cause collateral thermal damage to surrounding healthy oral tissues. Accordingly, oxygen-independent strategies that augment tumor killing under mild photothermal conditions may provide an effective means of addressing these therapeutic challenges in OSCC.

As an emerging therapeutic modality, thermodynamic therapy (TDT) based on thermally decomposable azo compounds, such as 2,2′-azobis[2-(2-imidazolin-2-yl)propane] dihydrochloride (AIPH), has shown considerable potential for cancer treatment. Unlike oxygen-dependent PDT, AIPH undergoes symmetric cleavage upon thermal stimulation to generate highly reactive alkyl radicals in an oxygen-independent manner. These radicals can directly attack biomacromolecules (such as DNA, lipids, and proteins) to induce potent oxidative stress and subsequent cell death within the severely hypoxic OSCC microenvironment [13,14]. Nevertheless, the therapeutic application of free AIPH remains limited by several challenges. First, due to its inherent thermal instability, AIPH can spontaneously produce toxic free radicals prematurely at the physiological temperature of 37 °C, potentially causing premature damage to surrounding healthy tissues [15]. Second, as a small-molecule compound, AIPH may undergo rapid clearance and metabolism in vivo, leading to insufficient accumulation at the tumor site. Therefore, developing an advanced delivery system capable of efficiently encapsulating AIPH and achieving its controlled release is of paramount importance for enhancing therapeutic efficacy and minimizing adverse effects.

Currently, various nanoplatforms have been explored for AIPH delivery, where AIPH is commonly loaded through physical adsorption. For instance, Guo et al. engineered a multifunctional nanoplatform (termed GZABEF) for hypoxic tumor therapy, where AIPH was physically embedded within the porous architecture of a zeolitic imidazolate framework-8 (ZIF-8) shell. Upon 1060 nm laser irradiation, the hyperthermia induced by GZABEF promoted the breakdown of AIPH to enable the efficient generation of alkyl radicals. To prevent radical scavenging by glutathione (GSH) within the tumor, this nanoplatform also incorporated GSH-regulating components to ensure the maximal accumulation of AIPH-derived alkyl radicals [16]. Similarly, Zhao et al. developed a copper-based nanoplatform (GAPSH) that incorporated AIPH into hollow mesoporous Cu_9_S_8_ nanoparticles for photothermal-triggered TDT, demonstrating the potential of nanocarrier-mediated AIPH delivery for hypoxic tumor therapy [17]. Although these delivery strategies employing nanocarriers have optimized the therapeutic efficacy of AIPH, there are still some critical issues that need to be addressed. First, the drug loading capacity of some nanocarriers may be insufficient for achieving the desired therapeutic dose of AIPH [18]. Second, the relatively weak physical adsorption of AIPH onto nanocarriers often causes premature leakage before reaching the target sites [19,20]. Most importantly, it is challenging for nanocarriers to achieve precise spatiotemporal synchronization between photothermal agents and thermodynamic sensitizers, which significantly undermines their synergistic therapeutic efficacy. Consequently, developing an advanced delivery platform capable of high drug loading, localized retention, and coordinated spatiotemporal co-localization of multiple therapeutic agents is highly desirable.

**Scheme 1:**
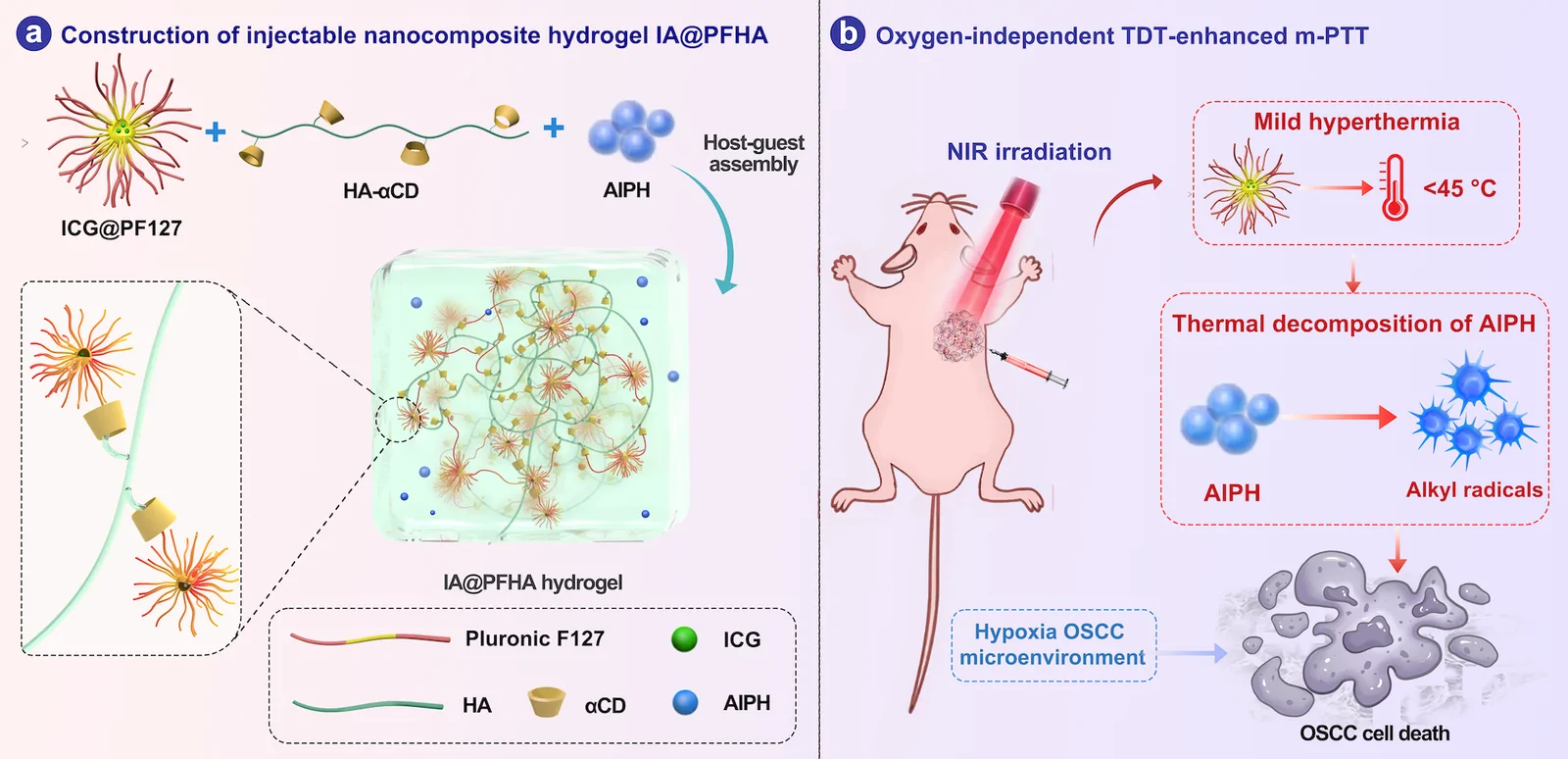
Schematic illustration of (**a**) the construction of the injectable nanocomposite hydrogel IA@PFHA and (**b**) the underlying tumor killing mechanism of IA@PFHA via oxygen-independent TDT-enhanced m-PTT for hypoxic OSCC.

In view of the above considerations, we developed a novel injectable nanocomposite hydrogel (denoted as IA@PFHA) based on a polypseudorotaxane architecture to enhance mild-temperature photothermal therapy (m-PTT) for OSCC via oxygen-independent TDT. As illustrated in Scheme 1, indocyanine green (ICG)-loaded nanoparticles (ICG@PF127) were co-assembled with *α*-cyclodextrin-modified hyaluronic acid (HA-*α*CD), with AIPH incorporated as an oxygen-independent radical initiator, to form the physically cross-linked IA@PFHA network. The gelation was primarily driven by host–guest inclusion between the surface-exposed poly(ethylene oxide) (PEO) segments of ICG@PF127 micelles and the *α*-cyclodextrin (*α*CD) cavities on the HA-*α*CD backbone. Such a physically cross-linked nanocomposite hydrogel not only ensures the stable localized co-retention of ICG and AIPH, but also endows the system with favorable shear-thinning properties, which facilitates minimally invasive injection and adaptation to the irregular anatomical contours of OSCC lesions. Upon near-infrared (NIR) irradiation, ICG endows the IA@PFHA hydrogel with mild hyperthermia (<45 °C), which promotes AIPH-derived alkyl radical generation in an oxygen-independent manner, thereby enhancing tumor cell killing under hypoxic conditions. Collectively, IA@PFHA integrates a physically cross-linked polypseudorotaxane network with NIR-triggered m-PTT and oxygen-independent radical generation, providing an injectable and hypoxia-tolerant therapeutic platform for enhanced OSCC treatment.

## 2. Materials and Methods

### 2.1. Chemicals and Bioreagents

All chemicals and bioreagents were obtained from commercial sources and used as received. Pluronic F127 and αCD were purchased from Sigma-Aldrich (St. Louis, MO, USA). HA was obtained from Yuan Ye Biological Technology Co., Ltd. (Shanghai, China). ICG was purchased from Dalian Meilun Biotech Co., Ltd. (Dalian, China). AIPH, 1-(3-dimethylaminopropyl)-3-ethylcarbodiimide hydrochloride (EDC·HCl), N-hydroxysulfosuccinimide sodium salt (Sulfo-NHS), 2-(N-morpholino)ethanesulfonic acid (MES), and 2,2′-azino-bis(3-ethylbenzothiazoline-6-sulfonic acid) diammonium salt (ABTS) were supplied by Bailingwei Technology Co., Ltd. (Beijing, China). Pyridine, p-toluenesulfonyl chloride (TsCl), and phthalimide were purchased from Aladdin Biotechnology (Shanghai, China). Hydrazine hydrate was provided by the Damao Chemical Reagent Factory (Tianjin, China). All organic solvents were bought commercially and used without further purification. Dulbecco’s Modified Eagle Medium (DMEM), α-Minimum Essential Medium (α-MEM), penicillin-streptomycin, and trypsin/ethylenediaminetetraacetic acid (EDTA) were purchased from Gibco (Grand Island, NY, USA). Fetal bovine serum (FBS) was purchased from Vivacell (Shanghai, China). The CCK-8 kit was purchased from APExBIO (Houston, TX, USA). The Calcein-AM/PI Cell Viability/Cytotoxicity Assay Kit was obtained from Beyotime Biological Reagent Co., Ltd. (Shanghai, China). DCFH-DA was provided by MedChemExpress (Shanghai, China).

### 2.2. Preparation and Characterization of ICG@PF127 Nanoparticles

ICG@PF127 nanoparticles were prepared by an emulsion-solvent evaporation method. Briefly, ICG was dissolved in 1.0 mL of chloroform at a concentration of 1.5 mg/mL. Pluronic F127 was dissolved in 5.0 mL of deionized water at a concentration of 2 wt%. Then, the ICG solution was added to the Pluronic F127 solution and subjected to ultrasonication (125 W, on/off cycle: 5 s/5 s, 2 min). Chloroform was removed by evaporation with magnetic stirring at room temperature. The unencapsulated ICG was separated using 3 kDa molecular weight cut off (MWCO) ultrafiltration tubes (Millipore, Burlington, MA, USA) at 6000 rpm for 60 min, and its concentration in the filtrate was subsequently quantified using a Hitachi UH5700 ultraviolet-visible (UV-Vis) spectrophotometer (Tokyo, Japan). The entire procedure was performed under light-protected conditions. The EE was calculated using Equation (1) and the LC using Equation (2).

(1) $$\mathrm{EE}\left(\%\right)=\frac{M_{\mathrm{ICG},\mathrm{added}}-M_{\mathrm{ICG},\mathrm{Unencapsulated}}}{M_{\mathrm{ICG},\mathrm{added}}}\times 100\%$$

(2) $$\mathrm{LC}\left(\%\right)=\frac{M_{\mathrm{ICG},\mathrm{added}}-M_{\mathrm{ICG},\mathrm{Unencapsulated}}}{M_{\mathrm{Nanoparticles}}}\times 100\%$$

The hydrodynamic diameter and polydispersity index (PDI) were determined using dynamic light scattering (DLS) (Zetasizer Nano ZS, Malvern Instruments, Malvern, UK). The morphology of ICG@PF127 nanoparticles was observed via a transmission electron microscope (TEM) (TALOS F200X, Thermo Fisher Scientific, Waltham, MA, USA). The UV-visible absorption spectra of the nanoparticles were measured by a HITACHI UV-Vis spectrophotometer.

### 2.3. Synthesis of HA-αCD Conjugate

The HA-αCD conjugate was synthesized by coupling NH_2_-αCD to the carboxyl groups of HA via Ean DC/NHS coupling reaction. First, αCD was reacted with TsCl to afford 6-Ts-αCD. Then, 6-Ts-αCD was reacted with phthalimide under alkaline conditions to introduce the protecting group. Thereafter, hydrazinolysis was carried out using hydrazine hydrate to remove the protecting group, thereby obtaining the aminated product NH_2_-αCD. The detailed procedures for the synthesis of 6-Ts-αCD and NH_2_-αCD are provided in the Supplementary Information. The detailed synthesis steps for HA-αCD are as follows. Briefly, HA (1000 mg, 0.03 mmol) was dissolved in 100 mL of MES buffer solution (pH 5.0) and stirred for 30 min. Next, EDC·HCl (1650 mg, 8.61 mmol) and Sulfo-NHS (1000 mg, 4.61 mmol) were added and allowed to react for 1 h at room temperature. Then, the pH of the solution was adjusted to 7.2 using 0.1 M phosphate-buffered saline (PBS, pH 7.4). Subsequently, NH_2_-αCD (500 mg, 0.51 mmol) was added, and the reaction mixture was stirred for another 24 h with nitrogen protection. The resulting solution was poured into a dialysis membrane (3500 Da MWCO) and dialyzed against deionized water for 5 days. Finally, HA-αCD was obtained via lyophilization. The chemical structure of HA-αCD and the degree of substitution of αCD were determined by ^1^H NMR.

### 2.4. Preparation and Characterization of the IA@PFHA Hydrogel

The hydrogel network was constructed through “host–guest” inclusion complexation between αCD moieties on the HA-αCD backbone and PEO segments of the ICG@PF127 nanoparticles. Initially, powdered HA-αCD was dissolved in deionized water at concentrations of 5, 10, 15, and 20 wt% and allowed to stand at room temperature for 8 h. Subsequently, AIPH was introduced to the solution at a final concentration of 12.5 mg/mL, followed by the addition of ICG@PF127 nanoparticles at a fixed concentration of 13.3 wt%. The mixture was thoroughly mixed and then conditioned at room temperature for gelation. The gelation times were estimated by visual inspection (the vial-tilting method), defined as the time at which the hydrogel precursor no longer flowed down for at least 2 min after tilting the vial. The morphology of the hydrogel was observed via scanning electron microscopy (SEM, TM4000plus, Hitachi, Tokyo, Japan). Prior to imaging, the sample was flash-frozen in liquid nitrogen, lyophilized, and sputter-coated with gold. The injectability of the hydrogel was assessed by its ability to pass through a 22-gauge needle. The rheological behavior of the hydrogel was evaluated using an Anton Paar rheometer (MCR302, Graz, Austria). The storage modulus (G′) and loss modulus (G″) were measured in oscillatory mode at 25 °C. Frequency sweep measurements were conducted by setting the constant shear strain (1%) and angular frequency range (0.1–100 rad/s). Further, strain sweep for the hydrogel was performed at angular frequency of 1 rad/s, with the strain ranging from 0.1% to 100%.

### 2.5. Photothermal Behavior of the Hydrogel and Generation of Free Radicals

The photothermal behaviors of the IA@PFHA hydrogel were investigated using an 808 nm NIR laser (Changchun Radium Photoelectric Co., Ltd., Changchun, China). Briefly, a total volume of 0.5 mL of sample was placed in a 48-well plate and then irradiated with the laser at varied power densities (0.5, 0.75, 1.0, 1.25, 1.5 W cm^−2^) for 10 min. The temperature changes were recorded using a digital thermometer (TES K-Type Digital Thermometer, Taiwan, China). In addition, the heating profiles of PBS (blank control), a mixture of free ICG and AIPH (IA Free), the hydrogel without AIPH (I@PFHA), and IA@PFHA were measured under identical conditions at an equivalent ICG concentration (20 μg/mL).

The generation of free radicals from the hydrogel subjected to thermal treatment was monitored via the oxidation of ABTS. Specifically, the IA@PFHA hydrogel (ICG concentration: 20 μg/mL; AIPH concentration: 250 μg/mL) was mixed with an equal volume (1:1, *v*/*v*) of ABTS aqueous solution (concentration: 2.0 mg/mL). Then, the mixtures were incubated in a water bath at 37 °C or 42 °C for 1, 2, and 4 h. The absorbance spectra (ranging from 400 to 900 nm) were recorded using a Tecan Spark microplate reader to monitor the production of ABTS^•+^. Furthermore, the radical generation profiles of IA Free and IA@PFHA hydrogel were measured under the identical conditions.

### 2.6. In Vitro Cytotoxicity Assessment of IA@PFHA Hydrogels

A cell counting kit (CCK-8) assay was employed to evaluate the cytocompatibility of the hydrogel with CAL 27 cells, a human tongue squamous carcinoma cell line, and MC3T3-E1 cells, a murine osteoblastic cell line. These cell lines were obtained from the Tianjin Key Laboratory of Oral and Maxillofacial Function Reconstruction at Tianjin Stomatological Hospital. CAL 27 and MC3T3-E1 cells were cultured in DMEM and α-MEM, respectively, both supplemented with 10% FBS and 1% penicillin-streptomycin. All cultures were maintained at 37 °C in a 5% CO_2_ atmosphere, with medium changes every 24 h and subculturing conducted at 70–80% confluence. For cytotoxicity evaluation, CAL 27 and MC3T3-E1 cells were seeded into 96-well plates (1.5 × 10^4^ cells/well) and cultured under either normoxic or hypoxic conditions established using an AnaeroPack (Mitsubishi Gas Chemical, Tokyo, Japan), according to the manufacturer’s instructions, for 24 h. The culture medium was then replaced with fresh medium containing different concentrations of IA@PFHA hydrogel (2, 4, 6, 8, and 10 mg/mL) and incubated for another 24 h. After washing twice with PBS, CCK-8 reagent diluted 10-fold with medium was added to each well. Following incubation for 4 h, the absorbance was measured at 450 nm. Finally, cell viability was calculated according to the Equation (3):

(3) Cell viability (%) = (A_treated_ − A_blank_)/(A_control_ − A_blank_) × 100%

where A_treated_ represents the absorbance of the experimental groups, A_blank_ represents the absorbance of the blank group, and A_control_ represents the absorbance of the control group.

### 2.7. In Vitro Anti-Tumor Efficacy

To explore the synergistic therapeutic efficacy of the IA@PFHA hydrogel, CAL 27 cells were plated in 48-well plates at a density of 5 × 10^4^ cells/well and cultured under both normoxic and hypoxic conditions using an anaerobic bag, as described in Section 2.6. Subsequently, the cells were treated with PBS, IA Free, I@PFHA, and IA@PFHA hydrogel, with the final concentrations of ICG and AIPH maintained at 20 μg/mL and 250 μg/mL, respectively. After incubating for 2 h, the designated groups were exposed to laser irradiation (808 nm, 0.75 W cm^−2^, 5 min). Following an additional incubation for 24 h, a CCK-8 assay was performed for quantitative detection of cell viability. The live/dead cells were distinguished using a Calcein-AM/PI Cell Viability/Cytotoxicity Assay Kit. Briefly, CAL 27 cells were seeded and treated following the same procedures as described before. At the end of the treatment period, the cells were co-stained with Calcein-AM and PI. Images were captured using an inverted fluorescence microscope (Eclipse, Ti-s, Nikon, Japan).

### 2.8. Intracellular Free Radical Detection

DCFH-DA was chosen to be the probe for the detection of intracellular free radical production, since its hydrolysis product could react with free radicals to form green fluorescent DCF. CAL 27 cells (1 × 10^5^) were seeded into 15 mm confocal dishes and cultured for 24 h. For normoxic conditions, cells were treated with PBS, IA Free, I@PFHA, and IA@PFHA hydrogel, with the final concentrations of ICG and AIPH maintained at 20 μg/mL and 250 μg/mL, respectively. Then, the culture medium was replaced with 300 μL of serum-free DMEM containing 10 μM DCFH-DA, and the cells were re-incubated for 30 min. Subsequently, the medium was replaced with fresh DMEM, and the designated groups were subjected to laser irradiation (808 nm, 0.75 W cm^−2^, 5 min). Afterward, the cells were washed twice with PBS and incubated with Hoechst 33342 for nuclear staining. The fluorescence signals of DCF and Hoechst 33342 were captured using Zeiss LSM 710 confocal laser scanning microscope (CLSM, Oberkochen, Germany). To investigate the influence of hypoxia on intracellular free radical generation, CAL 27 cells were cultured under oxygen-deprived conditions established using an AnaeroPack. Subsequently, the cells were treated with hydrogels and NIR laser irradiation, followed by the same staining and imaging protocols as described for the normoxic groups.

### 2.9. In Vivo Photothermal Performance and Synergistic Therapeutic Effect of IA@PFHA Hydrogel

The OSCC xenograft model was established by subcutaneously injecting 8 × 10^6^ CAL 27 cells (suspended in 100 μL of PBS) into the right dorsal flank of female BALB/c nude mice (4–5 weeks old). When the tumor volumes reached 90–120 mm^3^, the mice were randomly divided into four groups (n = 5). Subsequently, the mice were subjected to intratumoral injections of different formulations, including (1) PBS (Blank); (2) IA Free; (3) I@PFHA hydrogel; and (4) IA@PFHA hydrogel. The ICG and AIPH concentrations were maintained at 1 mg/mL and 12.5 mg/mL, respectively, in the corresponding formulations, with an injection volume of 25 μL per mouse. After 2 h, the mice were irradiated with an 808 nm laser at a power density of 1.0 W cm^−2^ for 10 min. Temperature changes at the tumor sites were recorded using an infrared thermal imaging camera. Both tumor volumes and mouse body weights were recorded every 4 days and calculated as 0.5 × length × width^2^. On day 28, the mice were euthanized, and the tumor tissues were excised, photographed, and weighed. The tumor inhibition rate (TIR) was calculated using the following equation: TIR (%) = (tumor weight of the control group − tumor weight of the treated group)/tumor weight of the control group × 100%.

### 2.10. Statistical Analysis

All experimental data were presented as mean ± standard deviation (SD). Statistical analysis was performed by one-way analysis of variance (ANOVA) or two-way ANOVA with GraphPad Prism 10.1.2 software. Significant differences were defined as follows: * *p* < 0.05, ** *p* < 0.01, *** *p* < 0.001, and **** *p* < 0.001 versus the control group; # *p* < 0.05, ## *p* < 0.01, ### *p* < 0.001 and #### *p* < 0.001 versus the blank group.

## 3. Results and Discussion

### 3.1. Characterization of ICG@PF127 Nanoparticles

**Figure 1:**
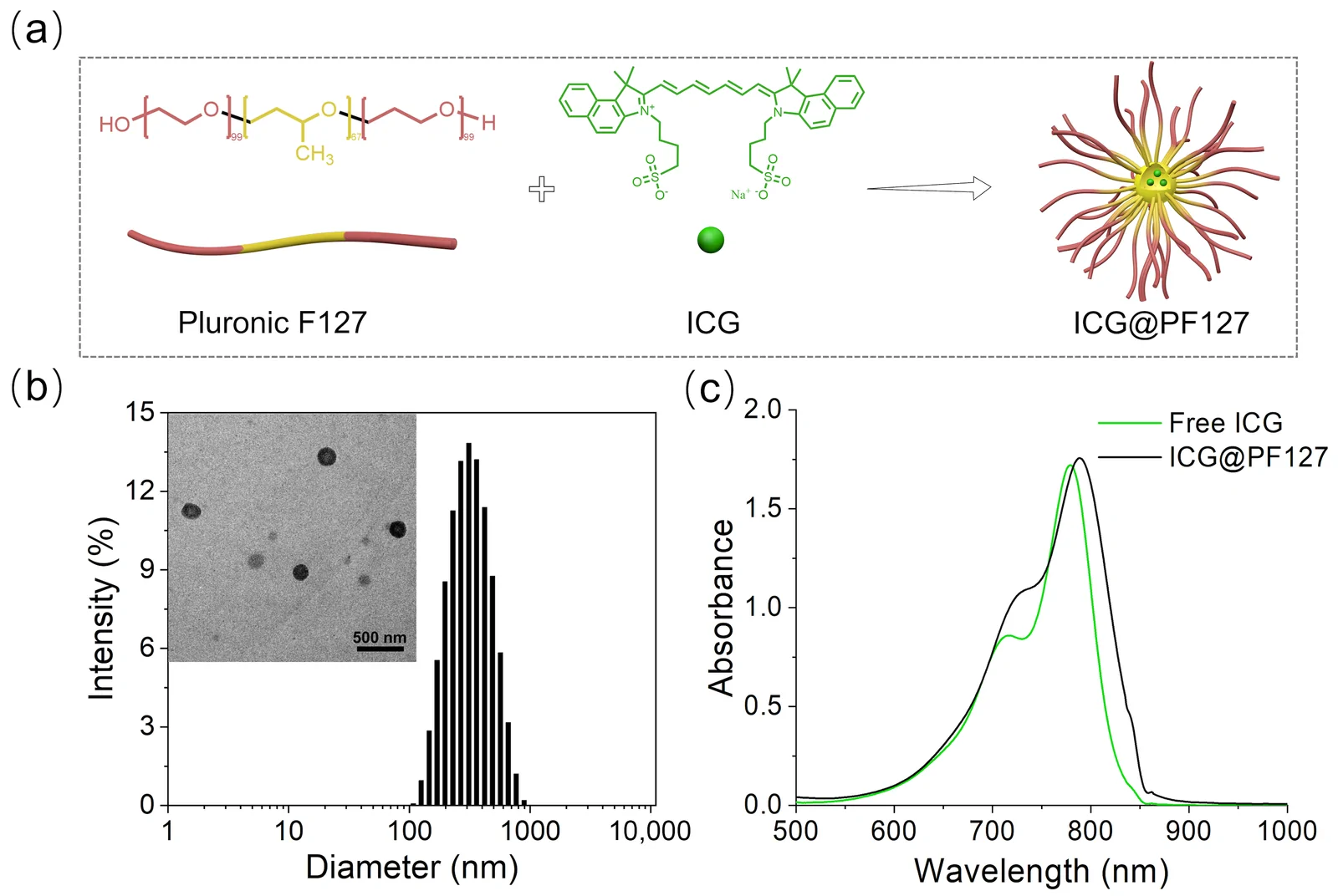
Fabrication and characterization of ICG@PF127 nanoparticles. (**a**) Schematic illustration of the formation of ICG@PF127 nanoparticles via the self-assembly of Pluronic F127 and ICG encapsulation; (**b**) hydrodynamic size distribution of ICG@PF127 nanoparticles measured by dynamic light scattering (DLS); inset: TEM image of ICG@PF127 nanoparticles (scale bar: 500 nm); (**c**) UV-Vis absorption spectra of free ICG and ICG@PF127 nanoparticles.

ICG@PF127 nanoparticles were constructed through self-assembly of the amphiphilic polymer Pluronic F127 in aqueous media. As illustrated in Figure 1a, the hydrophobic poly(propylene oxide) (PPO) segments of Pluronic F127 aggregate to form inner domains that accommodate ICG molecules. The encapsulation efficiency (EE) and loading capacity (LC) were determined to be 98.51% and 1.45%, respectively, indicating efficient incorporation of ICG into the Pluronic F127 micelles. As shown in Figure 1b, the hydrodynamic particle size was determined to be 218.87 ± 5.12 nm. The nanoparticles showed a polydispersity index (PDI) of 0.25 ± 0.05, consistent with a relatively homogeneous size distribution. The inset transmission electron microscope (TEM) image in Figure 1b shows that the ICG@PF127 nanoparticles exhibit spherical morphology without obvious aggregation. The average particle diameter, as measured from the TEM image using ImageJ (Image J 1.8.0), was 193.22 ± 13.17 nm. This value is smaller than the hydrodynamic size, which is likely attributable to the dehydration of nanoparticles during TEM sample preparation. Following self-assembly, the ICG absorption maximum shifted toward longer wavelengths, moving from 779 to 789 nm (Figure 1c), indicating the altered microenvironment of ICG after incorporation into F127 micelles.

### 3.2. Synthesis and Characterization of HA-αCD Conjugate

**Figure 2:**
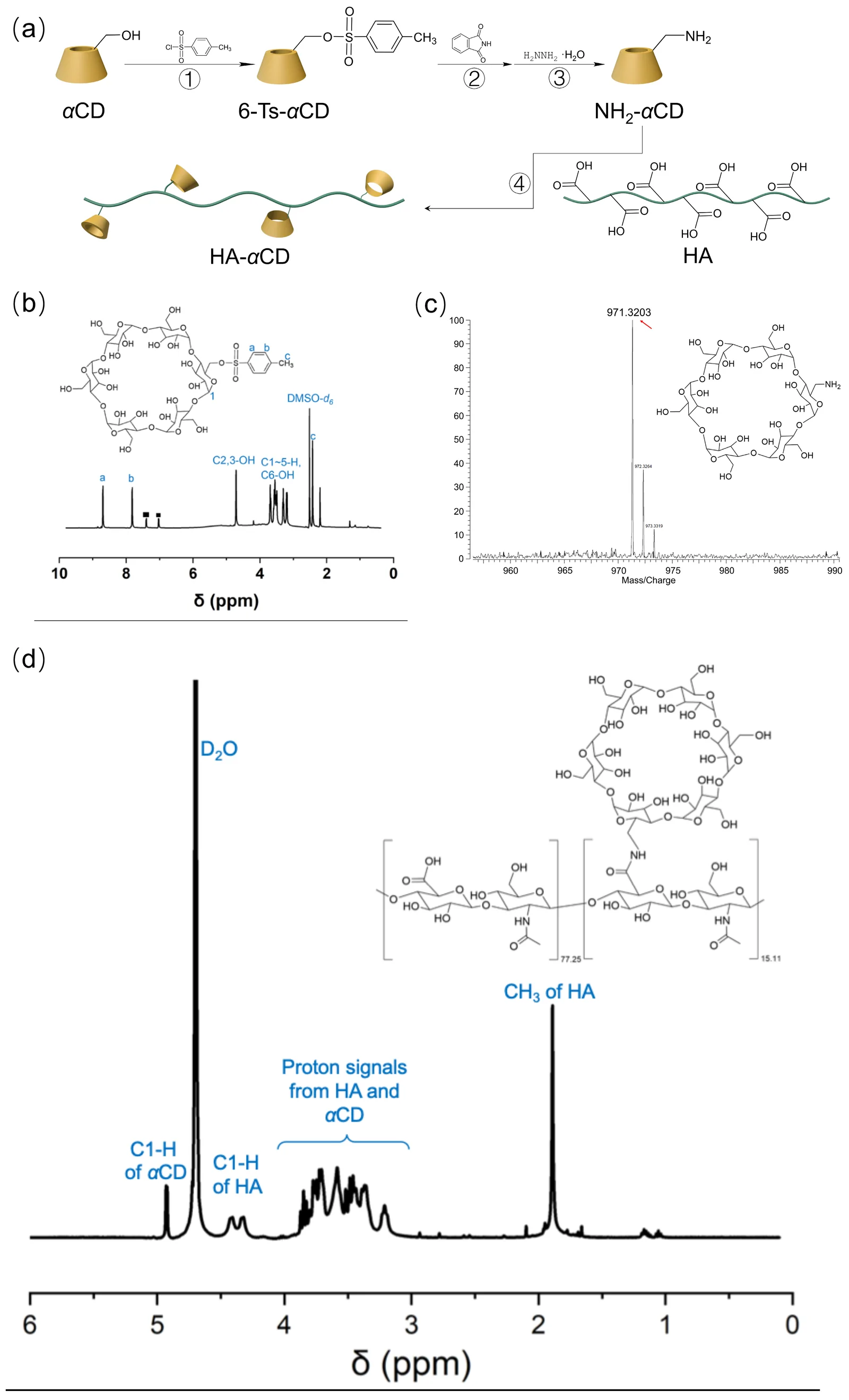
Synthesis and structural characterization of HA-αCD. (**a**) Schematic illustration of the synthetic route of HA-αCD, including the 6-Ts-αCD (①), phthalimide substitution (②), hydrazinolysis-mediated deprotection (③), and subsequent conjugation of NH_2_-αCD to HA via EDC/NHS coupling (④); (**b**) ^1^H NMR spectrum of 6-Ts-αCD; (**c**) mass spectrum of NH_2_-αCD; (**d**) ^1^H NMR spectrum of HA-αCD.

The synthesis of HA-αCD was performed via a multi-step pathway (Figure 2a), involving mono-tosylation of αCD to yield mono-tosylated α-cyclodextrin (6-Ts-αCD) (①), subsequent phthalimide substitution (②), and final hydrazinolysis-mediated deprotection to afford mono-amino-α-cyclodextrin (NH_2_-αCD) (③). Subsequently, NH_2_-αCD was conjugated to the hyaluronic acid (HA, Mw = 35 kDa) backbone via a 1-ethyl-3-(3-dimethylaminopropyl)carbodiimide hydrochloride/N-hydroxysuccinimide (EDC·HCl/NHS) coupling reaction, in which the amino groups of NH_2_-αCD reacted with the carboxyl groups of HA to form stable amide linkages (④). The chemical structure of 6-Ts-αCD was confirmed by ^1^H nuclear magnetic resonance (^1^H NMR). As shown in Figure 2b, 6-Ts-αCD exhibited signals in the range of 5.79–2.66 ppm, corresponding to the glucopyranose units (H1–H6) of αCD. Two distinct doublets in the downfield region at 8.69 and 7.82 ppm were assigned to the aromatic protons of the p-toluenesulfonyl group, alongside a singlet at 2.40 ppm corresponding to the methyl protons of p-toluenesulfonyl moiety. These features provided conclusive evidence for the successful substitution of the p-toluenesulfonyl group onto the αCD backbone. The intermediate 6-Ts-αCD was then converted to NH_2_-αCD, according to a previous report. Mass spectrometry (MS) was used to identify the product NH_2_-αCD. As illustrated in Figure 2c, an ion peak at m/z 971.32 (indicated by the red arrow) was observed, which was in accordance with its theoretical molecular weight calculated for [M]^+^ (971.85). Finally, the obtained NH_2_-αCD was conjugated onto the HA backbone. In the ^1^H NMR spectrum (Figure 2d), the typical peak for the methyl protons in HA was observed at 1.89 ppm, while the anomeric proton (CH-1) of the αCD clearly appeared at 4.92 ppm. The presence of both characteristic signals indicated the successful grafting of NH_2_-αCD onto the HA chain. Based on the integration areas of the two peaks, a substitution degree of 16.39% was obtained, corresponding to approximately 16.39 αCD units per 100 glucuronic acid–N-acetylglucosamine disaccharide units.

### 3.3. Characterization of IA@PFHA Hydrogel

IA@PFHA hydrogel was fabricated based on the non-covalent crosslinking between HA-αCD and ICG@PF127 nanoparticles. As illustrated in Section 3.1, the hydrophobic segments of Pluronic F127 encapsulate ICG to form the core, while the hydrophilic PEO segments extend outward to form the corona. The αCD moieties grafted onto the HA backbone possess hydrophobic cavities that spontaneously thread onto the PEO chains, forming polypseudorotaxanes. This host–guest interaction provides abundant physical crosslinking sites, resulting in the formation of a stable hydrogel network. To determine the optimal HA-αCD concentration for hydrogel formation, the HA-αCD concentration was varied while maintaining the ICG@PF127 concentration at 6.7 wt%. As shown in Figure S1, the solutions ceased to flow upon vial inversion at concentrations of 7.5 wt% and 10 wt%, confirming the formation of hydrogels. Consequently, the 7.5 wt% HA-αCD formulation was selected for subsequent experiments.

**Figure 3:**
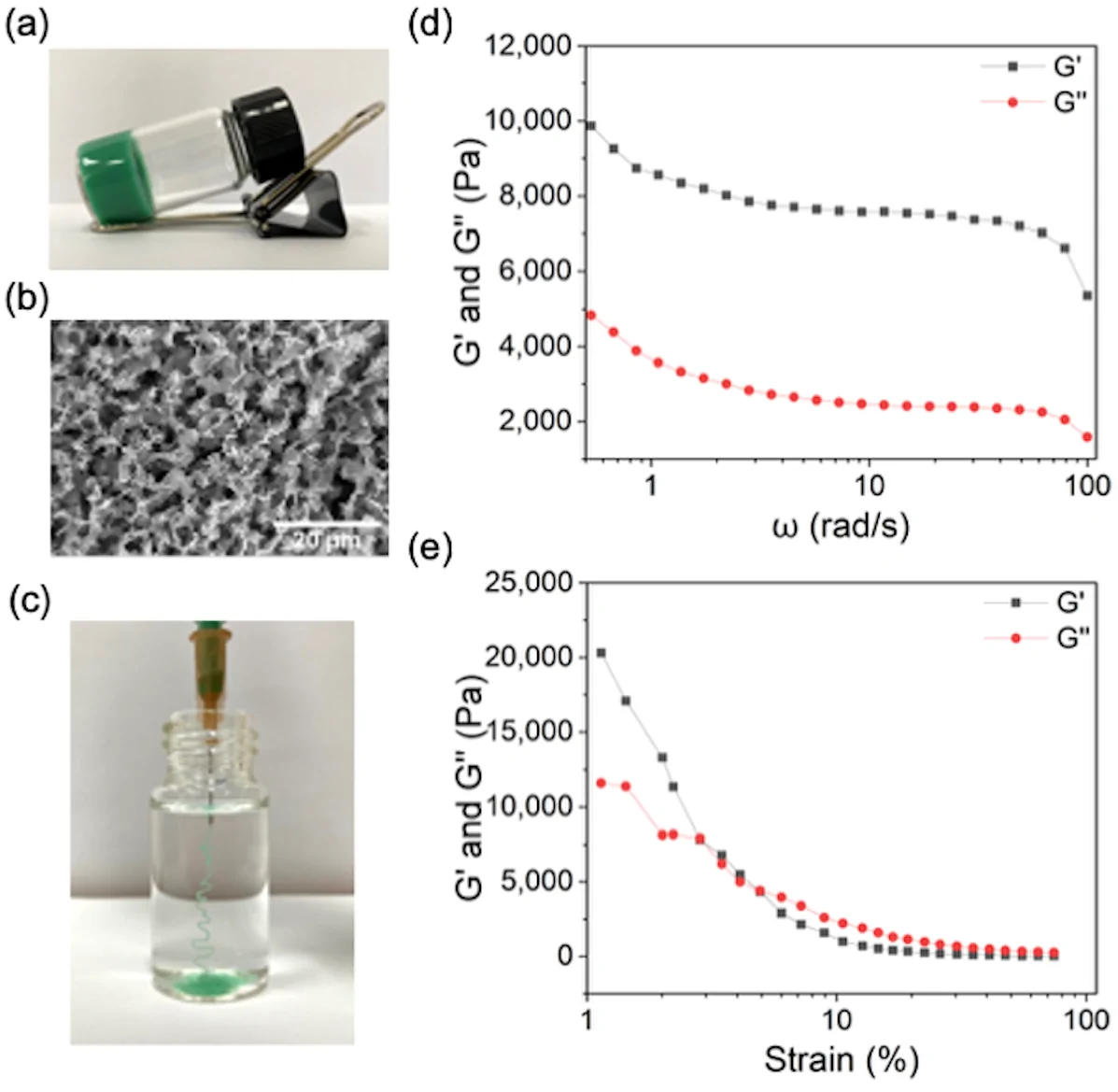
Characterization of the IA@PFHA hydrogel. (**a**) Photograph showing the gel state of IA@PFHA hydrogel after 72 h of storage at room temperature. (**b**) SEM image of IA@PFHA hydrogel showing its porous microstructure (scale bar: 20 μm). (**c**) Photograph demonstrating the injectability of the IA@PFHA hydrogel. (**d**) Frequency sweep of IA@PFHA hydrogel at a constant strain of 1%. (**e**) Strain sweep of IA@PFHA hydrogel at a fixed frequency of 1 Hz over a strain range of 0.1–100%.

Building upon this optimal formulation, the gelation time for this system was approximately 30 min (Figure S2). More importantly, the resulting IA@PFHA hydrogel could maintain a stable gel state at room temperature for at least 72 h (Figure 3a). Scanning electron microscopy (SEM) revealed that the freeze-dried hydrogel exhibited a well-organized, interconnected porous structure (Figure 3b). Furthermore, the IA@PFHA hydrogel demonstrated excellent injectability, as it could be extruded through a 22-gauge needle into the water without disintegration, highlighting its potential as an injectable platform for minimally invasive delivery (Figure 3c). Rheological experiments were carried out to assess the viscoelasticity of the hydrogel. As presented in Figure 3d, across the entire angular frequency range, the storage modulus (G’) consistently remained higher than the loss modulus (G″), indicating the formation of a stable crosslinked network. The strain sweep (Figure 3e) demonstrated the shear-thinning property of the hydrogel. Both G’ and G″ displayed a decreasing trend as strain increased, with a more pronounced decline in G’. At the crossover point (G′ = G″), the network underwent a gel-to-sol transition, indicating disruption of the physically crosslinked network under high strain and supporting the favorable injectability of the hydrogel.

### 3.4. Photothermal Behavior of the Hydrogel and Generation of Free Radicals

**Figure 4:**
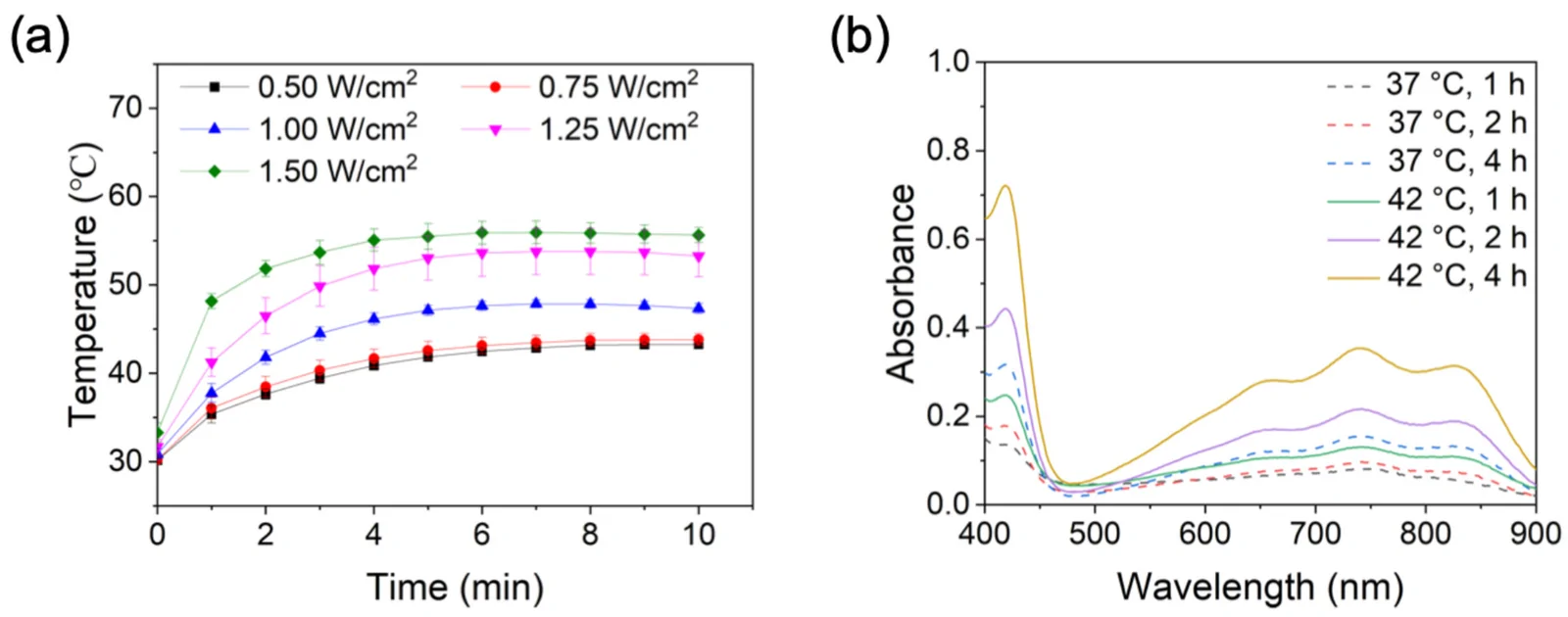
Photothermal performance and free radical generation capability of the IA@PFHA hydrogel. (**a**) Temperature elevation curves of the hydrogel under 808 nm laser irradiation at different power densities. (**b**) UV-Vis absorption spectra of ABTS^•+^ after incubation of the hydrogel with ABTS solution at 37 °C or 42 °C for 1, 2, and 4 h. Data are presented as mean ± standard deviation (SD) (*n* = 3).

To achieve oxygen-independent TDT, the photothermal effect of the IA@PFHA hydrogel should first provide sufficient heat to trigger the thermal decomposition of AIPH. Therefore, its photothermal performance was investigated by recording real-time temperature changes under 808 nm laser irradiation. As shown in Figure 4a, when irradiated at different power densities, the temperature of the hydrogel reached 41.8 °C and 42.9 °C within 5 min at 0.5 and 0.75 W cm^−2^, respectively. However, escalating the power density to 1.0 W cm^−2^ rapidly elevated the temperature above 47.6 °C, thereby exceeding the optimal therapeutic window for m-PTT (typically 42–45 °C). Based on these findings, 0.75 W cm^−2^ was selected for subsequent in vitro experiments. Building upon the favorable photothermal performance of the IA@PFHA hydrogel, we further assessed the capability of the encapsulated AIPH to undergo thermally induced radical generation. This process was quantified via a 2,2′-azino-bis(3-ethylbenzothiazoline-6-sulfonic acid) diammonium salt (ABTS) assay, which relies on the radical-induced oxidation of colorless ABTS into the green ABTS^•+^, a chromophore exhibiting a characteristic absorbance peak at 734 nm. As depicted in Figure 4b, free radical generation by the IA@PFHA hydrogel exhibited a temperature-dependent profile. At 37 °C, the absorbance of ABTS^•+^ remained minimal, showing negligible change throughout the 4 h incubation period. Conversely, at the mild photothermal temperature (44 °C), the IA@PFHA hydrogel induced a robust increase in ABTS^•+^ absorbance, significantly surpassing the levels observed at 37 °C across all corresponding time points. These findings indicate that, while negligible premature decomposition of AIPH may occur at physiological temperature, substantial radical generation was promoted upon reaching the mild photothermal temperature (44 °C), underscoring the temperature-responsive radical-generating capability of the hydrogel for TDT.

### 3.5. In Vitro Cytocompatibility of IA@PFHA Hydrogel

**Figure 5:**
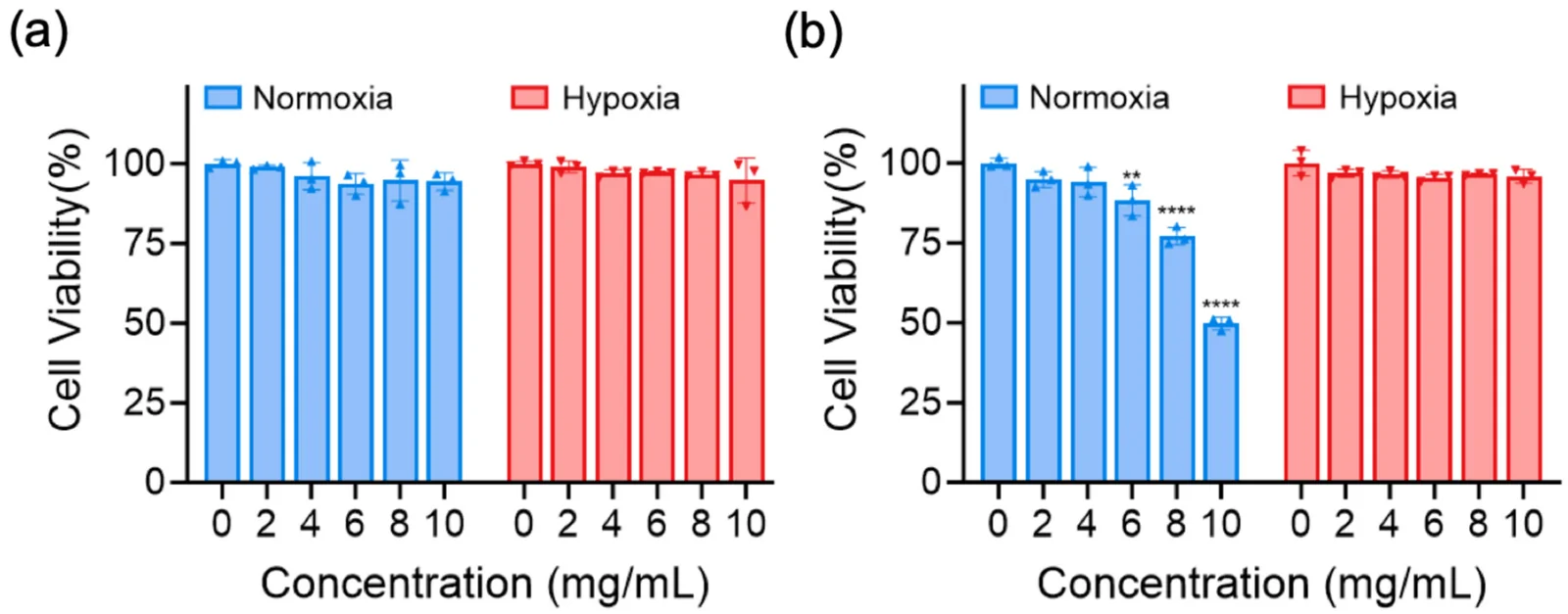
In vitro cytocompatibility of IA@PFHA hydrogel. (**a**) Cell viability of CAL 27 cells after treatment with IA@PFHA hydrogel under normoxic and hypoxic conditions. (**b**) Cell viability of MC3T3-E1 cells treated with IA@PFHA hydrogel under normoxic or hypoxic conditions. Statistical analysis was performed using one-way ANOVA, and statistical significance was determined relative to the blank control group. ** *p* < 0.01, **** *p* < 0.0001.

Excellent biocompatibility of hydrogels is a fundamental prerequisite for their application in tumor therapy. To this end, the in vitro cytotoxicity of the IA@PFHA hydrogel was assessed against CAL 27 oral squamous carcinoma cells and MC3T3-E1 cells (a representative normal cell line) under both normoxic and hypoxic conditions. As shown in Figure 5a, the viability of CAL 27 cells remained consistently above 95% at concentrations up to 10 mg/mL, regardless of oxygen levels, indicating negligible basal cytotoxicity of the IA@PFHA hydrogel toward CAL 27 cell line. Regarding the MC3T3-E1 cells (Figure 5b), cell viability remained above 75% even at a concentration of 8 mg/mL under normoxic conditions, while it exceeded 95% under hypoxic conditions. This enhanced cell viability under hypoxic conditions may be partially associated with the adaptive response of MC3T3-E1 cells to oxygen deficiency. Previous studies have demonstrated that hypoxia-induced activation of the hypoxia-inducible factor 1-α/metastasis-associated gene 1 (HIF-1α/MTA 1) signaling pathway can promote MC3T3-E1 proliferation, which likely contributes to the improved cellular tolerance observed under hypoxic conditions [21]. Overall, these results revealed that the IA@PFHA hydrogel exerted low cytotoxicity towards either tumor or normal cells within the tested concentration range under both normoxic and hypoxic conditions. Accordingly, the hydrogel concentration used in subsequent experiments was maintained within this safe range.

### 3.6. In Vitro Anti-Tumor Efficacy of the IA@PFHA Hydrogel

**Figure 6:**
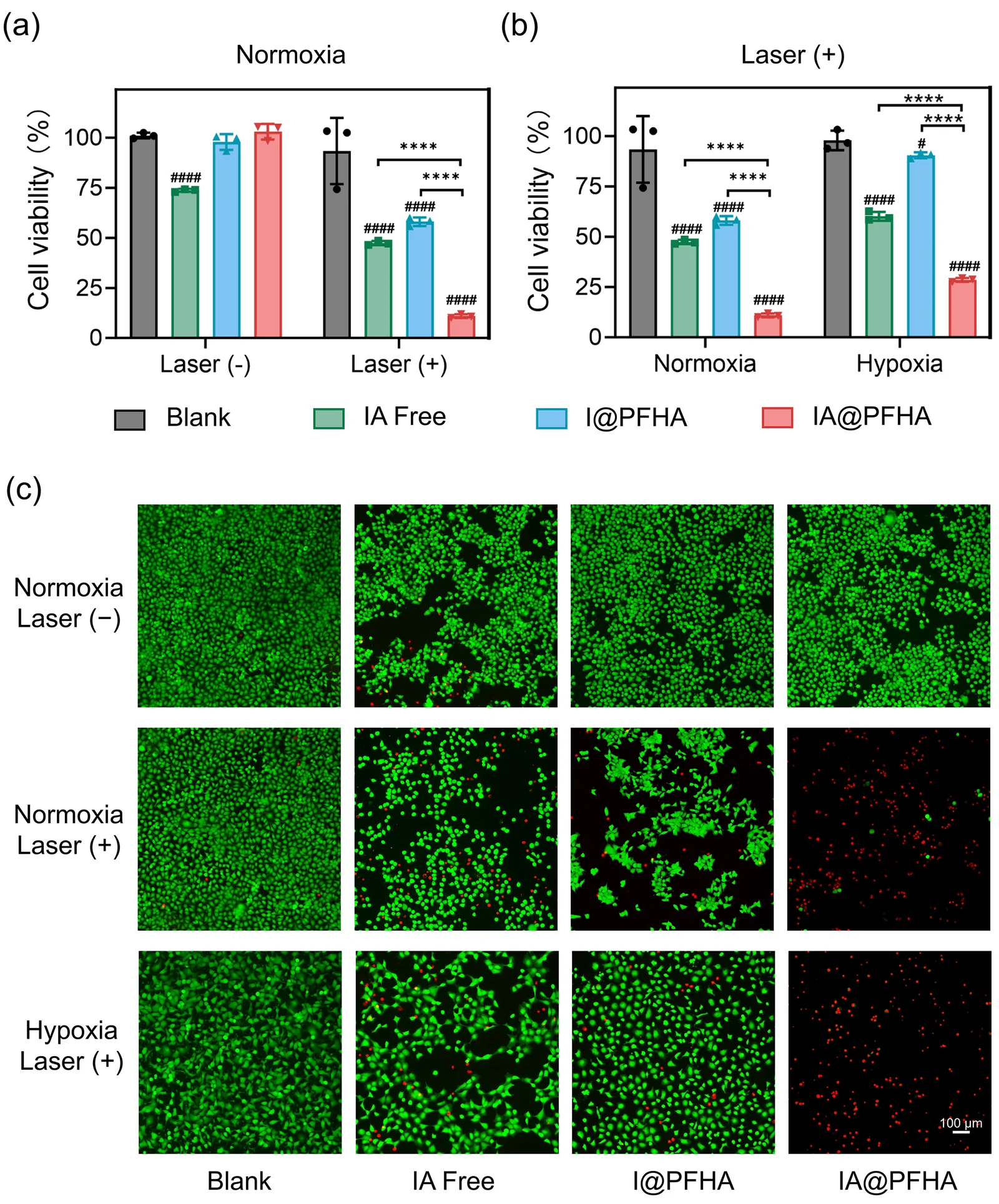
In vitro anti-tumor efficacy of IA@PFHA hydrogel toward CAL 27 cells. (**a**) CCK-8 assay under normoxic condition comparing laser-irradiated (Laser (+)) and non-irradiated (Laser (−) groups to evaluate the therapeutic effect triggered by 808 nm laser irradiation. (**b**) CCK-8 assay in which all groups received 808 nm laser irradiation (0.75 W cm^−2^, 5 min), comparing normoxic and hypoxic conditions to evaluate the influence of oxygen availability on the therapeutic efficacy. (**c**) Representative calcein-AM/PI live/dead fluorescence images of CAL 27 cells treated with Blank, IA Free, I@PFHA, or IA@PFHA under normoxic conditions without laser irradiation or under normoxic and hypoxic conditions with 808 nm laser irradiation. Green fluorescence indicates viable cells, whereas red fluorescence indicates dead cells. Scale bar: 100 μm. Data are presented as mean ± SD (n = 3). Statistical analysis was performed using two-way ANOVA followed by Šídák’s multiple comparisons test. # *p* < 0.05, #### *p* < 0.0001 versus the Blank group; **** *p* < 0.0001.

The in vitro anti-tumor efficacy of the IA@PFHA hydrogel was evaluated via cell counting kit (CCK-8) assays under both normoxic and hypoxic conditions. Under normoxic conditions, the IA@PFHA hydrogel showed negligible effects on cell viability in the absence of laser irradiation, with a cell viability of 102.5% (Figure 6a), indicating negligible basal cytotoxicity, consistent with the results described above. In contrast, the mixture of free ICG and AIPH group (IA Free) exhibited a 26.3% reduction in cell viability, which may be attributed to the partial thermal decomposition of AIPH and subsequent low-level radical generation at physiological temperature (Figure 4b). The IA@PFHA hydrogel effectively alleviated this nonspecific toxicity, likely due to the confinement of AIPH within the hydrogel network, which may limit radical diffusion and cellular exposure. When exposed to 808 nm laser irradiation, all treatment groups exhibited a significant decrease in cell viability compared with the blank group. Of note, the IA@PFHA hydrogel achieved the greatest reduction in cell viability, significantly outperforming the IA Free and the hydrogel without AIPH (I@PFHA) groups. These results demonstrate that photothermally triggered alkyl radicals substantially enhance the anti-tumor efficacy of m-PTT. Considering the hypoxic nature of the tumor microenvironment, the therapeutic performance was further evaluated under hypoxic conditions (Figure 6b). Intriguingly, unlike the I@PFHA hydrogel, whose efficacy was markedly compromised under hypoxic conditions, the IA@PFHA hydrogel maintained comparable anti-tumor potency, highlighting its robust oxygen-independent therapeutic potential.

To further validate the cell-killing efficacy of the IA@PFHA hydrogel against CAL 27 cells, a calcein acetoxymethyl ester (calcein-AM)/propidium iodide (PI) staining assay was then performed to visualize the live (green) and dead cells (red) under an inverted fluorescence microscope. As shown in Figure 6c, under normoxic conditions without laser irradiation, nearly all CAL 27 cells treated with the hydrogel remained green. This visual evidence is highly consistent with the quantitative CCK-8 results, further confirming the negligible basal cytotoxicity of the IA@PFHA hydrogel. After laser irradiation under both normoxic and hypoxic conditions, almost all cells in the blank group remained viable, indicating that laser irradiation alone exerted no detectable phototoxicity toward CAL 27 cells. Concurrently, a significant decrease in cell viability was observed in the treatment groups. Importantly, the cell-killing efficiency of the I@PFHA group was significantly attenuated under hypoxic conditions compared to normoxic conditions, as evidenced by the increased green fluorescence. In stark contrast, green fluorescence was barely observed in the IA@PFHA group under both normoxic and hypoxic conditions. These findings are consistent with the results of the CCK-8 assay, further supporting the robust anti-tumor efficacy of the IA@PFHA hydrogel under both normoxic and hypoxic conditions.

### 3.7. Intracellular Free Radical Generation Triggered by IA@PFHA Hydrogel

**Figure 7:**
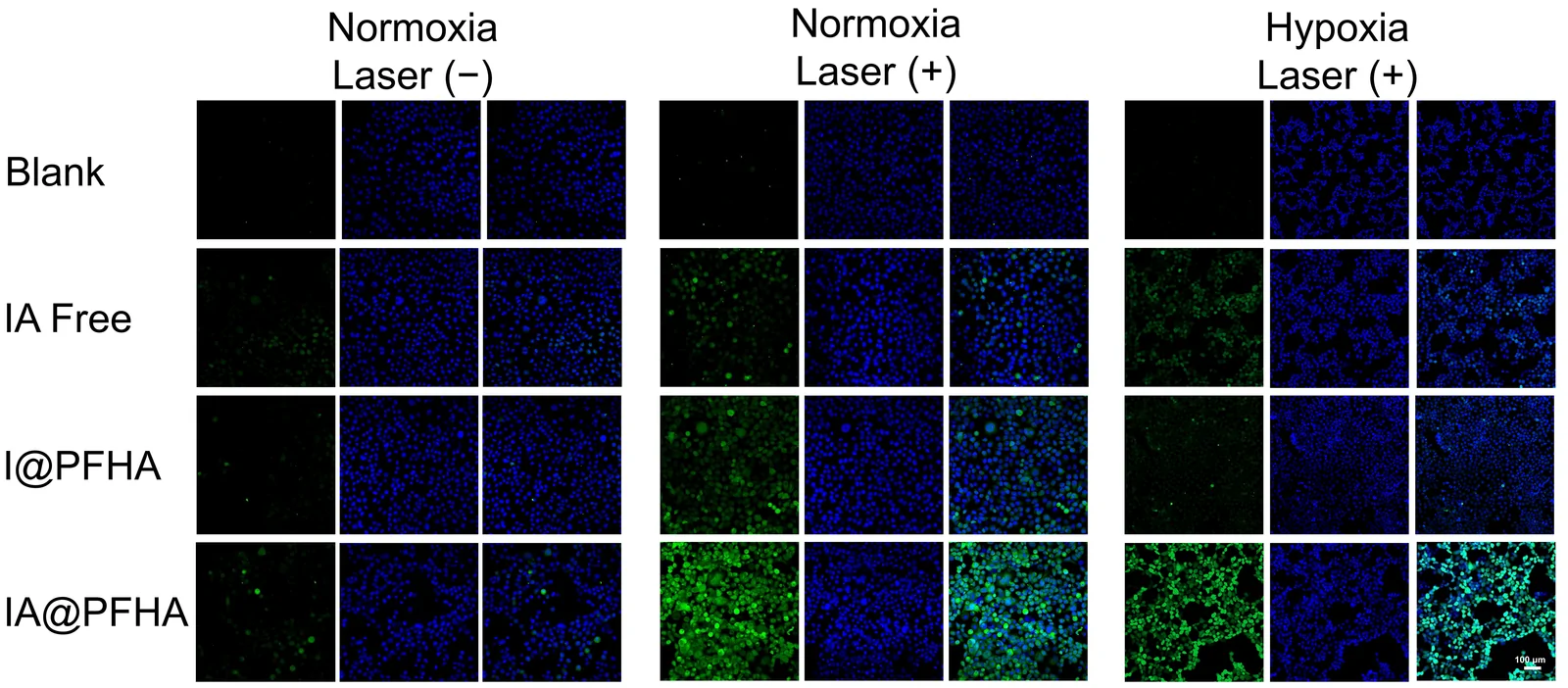
Intracellular free radical generation triggered by IA@PFHA hydrogel. Confocal laser scanning microscopy (CLSM) images of CAL 27 cells after different treatments. Intracellular reactive radical signals were detected using the DCFH-DA fluorescent probe (green DCF fluorescence) under normoxic and hypoxic conditions in Blank, IA Free, I@PFHA, and IA@PFHA groups. Cell nuclei were counterstained with Hoechst 33342 (blue fluorescence). The corresponding green, blue, and merged images are presented. Scale bar = 100 μm.

The IA@PFHA hydrogel was developed as a synergistic therapeutic platform aimed at enhancing the efficacy of m-PTT through TDT-derived free radical generation. To investigate the radical-generating capability of IA@PFHA and its potential application in hypoxic tumor therapy, intracellular reactive species production was evaluated using 2′,7′-dichlorodihydrofluorescein diacetate (DCFH-DA) as a fluorescent probe. DCFH-DA can be oxidized by reactive species to generate 2′,7′-dichlorofluorescein (DCF), producing green fluorescence. As shown in Figure 7, under normoxic conditions without laser irradiation, cells treated with the formulations without AIPH (I@PFHA) exhibited negligible fluorescence signals, whereas the AIPH-containing groups (IA Free and IA@PFHA) displayed weak fluorescence, indicating slight radical generation caused by the basal thermal decomposition of AIPH at physiological temperature (37 °C). Upon NIR irradiation, all treatment groups displayed enhanced fluorescence signals, indicating that mild photothermal heating enhanced intracellular radical generation. Notably, the IA@PFHA hydrogel-treated group exhibited the strongest fluorescence intensity among all tested formulations after laser irradiation. This improvement may be attributed to the nanocomposite hydrogel structure, in which ICG@PF127 nanoparticles and AIPH are simultaneously confined within the polypseudorotaxane network, which may promote the retention of AIPH and enhance its thermal activation. Importantly, similar fluorescence enhancement was observed under hypoxic conditions. Compared with the normoxic environment, IA@PFHA maintained pronounced radical generation after NIR irradiation, demonstrating that the AIPH-mediated radical production mechanism remains effective under oxygen-deficient conditions.

### 3.8. In Vivo Synergistic Therapeutic Efficacy of IA@PFHA Hydrogel

**Figure 8:**
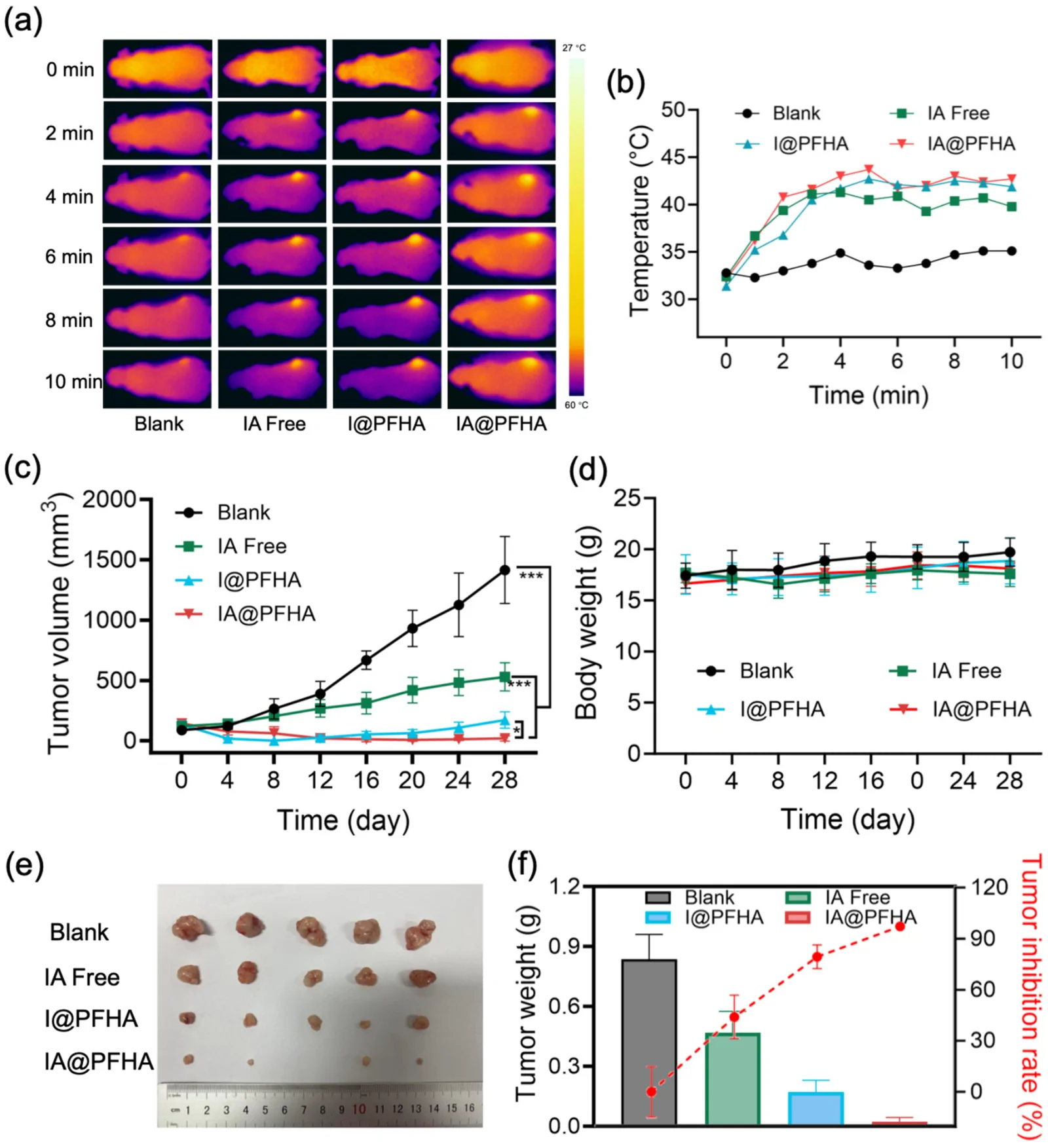
In vivo photothermal performance and enhanced therapeutic efficacy of the IA@PFHA hydrogel against OSCC. (**a**) Infrared thermal images and (**b**) corresponding temperature profiles of CAL 27 tumor-bearing mice from the Blank, IA Free, I@PFHA, and IA@PFHA groups during 808 nm laser irradiation (1 W cm^−2^, 10 min). (**c**) Tumor growth curves and (**d**) body weight changes of mice in each treatment group during the treatment period. (**e**) Representative photographs of excised tumors collected from each group at the end of the treatment. (**f**) Quantitative analysis of excised tumor weights and corresponding TIRs. Data are presented as mean ± SD. Statistical analysis was performed using one-way ANOVA, * *p* < 0.05, *** *p* < 0.001.

Building on the promising in vitro results, an orthotopic xenograft mouse model of OSCC was constructed to investigate the in vivo anti-tumor therapeutic efficacy of the IA@PFHA hydrogel. Tumor-bearing mice were randomly assigned to four groups: (1) Blank (PBS); (2) IA Free; (3) I@PFHA hydrogel; and (4) IA@PFHA hydrogel. After intratumoral administration, all groups were exposed to 808 nm laser irradiation (1 W cm^−2^) for 10 min. As shown in Figure 8a,b, infrared thermal imaging demonstrated that all ICG-containing formulations (IA Free, I@PFHA, and IA@PFHA) exhibited comparable photothermal profiles at the tumor sites. Specifically, the tumor temperatures rapidly increased to 41–43 °C within 3 min and subsequently stabilized below 45 °C throughout irradiation. Such mild hyperthermia falls within the intended temperature range for m-PTT and is sufficient to promote AIPH-mediated radical generation. In contrast, the PBS group showed only a slight temperature increase (~2.5 °C), demonstrating that 808 nm laser irradiation alone was insufficient to induce substantial local hyperthermia. Tumor growth was recorded every 4 days during the treatment period (Figure 8c). The PBS group exhibited rapid tumor progression, indicating that NIR irradiation alone produced minimal anti-tumor effects. Similarly, IA Free treatment showed only moderate tumor growth inhibition, which may be associated with the limited tumor retention of the free therapeutic components. In comparison, both hydrogel groups (I@PFHA and IA@PFHA) demonstrated significantly improved tumor suppression, highlighting the advantages of the hydrogel platform in enhancing local therapeutic efficacy. Notably, the IA@PFHA hydrogel achieved the most pronounced inhibition of tumor growth among all treatment groups, which can be attributed to the synergistic effect of NIR-triggered photothermal effect and AIPH-mediated radical generation within the hydrogel network. The enhanced therapeutic performance of IA@PFHA was further confirmed by ex vivo tumor imaging and tumor weight analysis (Figure 8e,f). The IA@PFHA-treated mice exhibited the smallest tumor size and the lowest tumor weight, corresponding to the highest tumor inhibition rate (TIR, approximately 97.2%). These results demonstrate that oxygen-independent TDT could enhance the therapeutic efficacy of m-PTT, which could potentially address hypoxia-associated limitations in OSCC treatment. Throughout the treatment period, the body weights of mice in all groups remained relatively stable (Figure 8d), indicating no obvious systemic toxicity of the IA@PFHA hydrogel. Collectively, these findings demonstrate that the injectable IA@PFHA hydrogel effectively integrates m-PTT heating with oxygen-independent AIPH-mediated radical generation, thereby enhancing anti-tumor efficacy against OSCC.

## 4. Conclusions

In summary, we developed an injectable nanocomposite hydrogel (IA@PFHA) based on a polypseudorotaxane host–guest cross-linked architecture. The dynamic and reversible supramolecular network endowed the hydrogel with favorable shear-thinning injectability and in situ gelation capability. Importantly, the three-dimensional network enabled the localized co-delivery of ICG and AIPH, facilitating their spatial colocalization. Upon 808 nm laser irradiation, ICG-mediated photothermal conversion generated mild hyperthermia that promoted AIPH decomposition and alkyl radical generation, thereby coupling photothermal therapy with oxygen-independent thermodynamic therapy (TDT) within a localized delivery system.

Unlike conventional high-temperature photothermal ablation, this strategy employed m-PTT to limit excessive thermal exposure and potential adverse effects. To enhance the therapeutic efficacy of m-PTT, AIPH was incorporated as a thermally activated radical generator whose decomposition does not require molecular oxygen as a reactant. This feature may help mitigate the limitations imposed by tumor hypoxia. In vivo experiments demonstrated that IA@PFHA achieved enhanced tumor inhibition compared with both the AIPH-free hydrogel and IA Free combination therapy with comparable photothermal effects, highlighting the contribution of hydrogel-mediated oxygen-independent TDT in overcoming hypoxia-associated therapeutic limitations.

Collectively, the findings from this study demonstrate an injectable nanocomposite hydrogel strategy that enhances m-PTT of OSCC through oxygen-independent TDT, providing a potential approach to overcome hypoxia-associated limitations in oral cancer treatment.

## Data Availability

The raw data supporting the conclusions of this article will be made available by the authors on request.

## Supplementary Materials

The following supporting information can be downloaded at: https://www.mdpi.com/article/10.3390/polym18212767/s1. Figure S1: Visual observation of the gelation behavior of IA@PFHA with different HA-αCD concentrations (2.5, 5.0, 7.5, and 10.0 wt%) using the vial inversion method; Figure S2: Visual observation of the gelation process of IA@PFHA at room temperature. The gelation time was recorded when the solution ceased to flow.

## References

[1] Jing F., Zhu L., Zhang J., Zhou X., Bai J., Li X., Zhang H., Li T. (2024). Multi-omics reveals lactylation-driven regulatory mechanisms promoting tumor progression in oral squamous cell carcinoma. Genome Biol..

[2] Zhou W., Feng Y., Lin C., Chao C.K., He Z., Zhao S., Xue J., Zhao X.-Y., Cao W. (2023). Yin Yang 1-Induced Long Noncoding RNA DUXAP9 Drives the Progression of Oral Squamous Cell Carcinoma by Blocking CDK1-Mediated EZH_2_ Degradation. Adv. Sci..

[3] Wang C.-I., Chen C.-Y., Chen T.-W., Cheng C.-C., Hong S.-W., Tsai T.-Y., Chang K.-P. (2025). PSMA2 promotes chemo- and radioresistance of oral squamous cell carcinoma by modulating mitophagy pathway. Cell Death Discov..

[4] Mrosk F., Vertic V., Richter M., Knödler L., Alfertshofer M., Hausmann F., Klinghammer K., Voss J.O., Doll C., Rendenbach C. (2025). Clinical and oncological outcome in locoregional advanced oral squamous cell carcinoma treated by primary chemoradiation. Oral Oncol..

[5] Fan Y., Cui C., Rosen C.J., Sato T., Xu R., Li P., Wei X., Bi R., Yuan Q., Zhou C. (2022). Klotho in Osx+-mesenchymal progenitors exerts pro-osteogenic and anti-inflammatory effects during mandibular alveolar bone formation and repair. Signal Transduct. Target. Ther..

[6] Tan B., Tang Q., Zhong Y., Wei Y., He L., Wu Y., Wu J., Liao J. (2021). Biomaterial-based strategies for maxillofacial tumour therapy and bone defect regeneration. Int. J. Oral Sci..

[7] Zhang W., Chen S., Bai Z., Gan M., Chen M., Zhang Y., Liu S., Liu D. (2024). Photodynamic Therapy for Oral Squamous Cell Carcinoma: Current Status, Challenges, and Prospects. Int. J. Nanomed..

[8] Li X., Hao M., Liu A., Li L., Nešić M.D., Yang B., Liu W., Lin Q. (2024). Dual-activity nanozyme as an oxygen pump to alleviate tumor hypoxia and enhance photodynamic/ NIR-II photothermal therapy for sniping oral squamous cell carcinoma. Acta Biomater..

[9] Yin Q., Zhang J., Zhang H., Gao J., Weng L., Liu T., Sun S., Yao Y., Chen X. (2024). Cascade Nanoreactor Employs Mitochondrial-Directed Chemodynamic and δ-ALA-Mediated Photodynamic Synergy for Deep-Seated Oral Cancer Therapy. Adv. Healthc. Mater..

[10] dos Santos M.S.C., Gouvêa A., de Moura L.D., Paterno L.G., de Souza P.E.N., Bastos A., Damasceno E.A.M., Veiga-Souza F.H., de Azevedo R.B., Báo S.N. (2018). Nanographene oxide-methylene blue as phototherapies platform for breast tumor ablation and metastasis prevention in a syngeneic orthotopic murine model. J. Nanobiotechnol..

[11] Shi F., Luo D., Zhou X., Sun Q., Shen P., Wang S. (2021). Combined effects of hyperthermia and chemotherapy on the regulate autophagy of oral squamous cell carcinoma cells under a hypoxic microenvironment. Cell Death Discov..

[12] Wan Y., Fu L.-H., Li C., Lin J., Huang P. (2021). Conquering the Hypoxia Limitation for Photodynamic Therapy. Adv. Mater..

[13] Li S., Yang F., Wang Y., Du T., Hou X. (2023). Emerging nanotherapeutics for facilitating photodynamic therapy. Chem. Eng. J..

[14] Mohanto N., Mondal H., Park Y.-J., Jee J.-P. (2025). Therapeutic delivery of oxygen using artificial oxygen carriers demonstrates the possibility of treating a wide range of diseases. J. Nanobiotechnol..

[15] Li J., Luo C., Sun T., Zhou Y., Huang X., Wu D., Luo X., Zeng C., Li H. (2024). Hypoxia-Specific Metal–Organic Frameworks Augment Cancer Immunotherapy of High-Intensity Focused Ultrasound. ACS Nano.

[16] Li M., Ye Z., Wang Y., Kong H., Zhou J., Cao J., Zhan H., Li Q., Peng X. (2025). Bidirectional pH-responsive O2@perfluorocarbon@cyanine dyes for cascade photothermal therapy and oxygen-releasing enhanced photodynamic tumor treatment. Chem. Eng. J..

[17] Guan M., Cheng K., Xie X.-T., Li Y., Ma M.-W., Zhang B., Chen S., Chen W., Liu B., Fan J.-X. (2024). Regulating copper homeostasis of tumor cells to promote cuproptosis for enhancing breast cancer immunotherapy. Nat. Commun..

[18] Wang C., Li Y., Yang W., Zhou L., Wei S. (2021). Nanozyme with Robust Catalase Activity by Multiple Mechanisms and Its Application for Hypoxic Tumor Treatment. Adv. Healthc. Mater..

[19] Hu H., Deng X., Song Q., Yang W., Zhang Y., Liu W., Wang S., Liang Z., Xing X., Zhu J. (2021). Mitochondria-targeted accumulation of oxygen-irrelevant free radicals for enhanced synergistic low-temperature photothermal and thermodynamic therapy. J. Nanobiotechnol..

[20] Li W., Li B., Wu B., Tian B., Chen X., Wang C., Hong W., Peng J. (2022). Free-Radical Cascade Generated by AIPH/Fe_3_O_4_-Coloaded Nanoparticles Enhances MRI-Guided Chemo/Thermodynamic Hypoxic Tumor Therapy. ACS Appl. Mater. Interfaces.

[21] Liu T., Zou W., Shi G., Xu J., Zhang F., Xiao J., Wang Y. (2015). Hypoxia-induced MTA1 promotes MC3T3 osteoblast growth but suppresses MC3T3 osteoblast differentiation. Eur. J. Med. Res..

