# Supplementary material for: Injectable Nanocomposite Hydrogel for Enhanced Mild Photothermal Therapy of Oral Cancer via Oxygen-Independent Thermodynamic Therapy
Source: Polymers (Basel). 2026 Nov 6;18(21):2767. doi: 10.3390/polym18212767 (PMC13766154; doi:10.3390/polym18212767)
Supplement: Supplementary file 1 [file polymers-18-02767-s001.zip › polymers-4605667-supplementary.pdf]

Supporting Information

Injectable Nanocomposite Hydrogel for Enhanced Mild Photothermal Therapy of Oral Cancer via Oxygen-Independent Thermodynamic Therapy

Wenjuan Chen [1,3,4,†,*], Hui Lv [2,3,4,†], Xiaoyan Zhang [1,3,4] and Jun Zhang [2,3,4,*]

1 Department of Central Laboratory, Tianjin Stomatological Hospital, School of Medicine, Nankai University, Tianjin 300041, China

2 Department of Oromaxillofacial-Head and Neck Oncology, Tianjin Stomatological Hospital, School of Medicine, Nankai University, Tianjin 300041, China

3 Tianjin Key Laboratory of Oral and Maxillofacial Function Reconstruction & Stomatology Institute of Nankai University, Tianjin 300041, China

* Correspondence: chen.wenjuan@nankai.edu.cn (W.C.); zhangjun66@tj.gov.cn (J.Z.)

Tel.: +86-22-27119191 (W.C. & J.Z.)

† These authors contributed equally to this work.

Contents:

1. Experimental procedures

1.1 Synthesis of mono-Tosylation *α*-cyclodextrin (Ts-*α*CD)

Ts-α-CD was synthesized through the selective tosylation of α-cyclodextrin (α-CD). First, *α*-cyclodextrin (5.0 g, 5.14 mmol) and pyridine (50 mL, 0.62 mol) were added to an eggplant flask and homogenized via sonication. The eggplant flask was immersed in an oil bath at 30 °C. Then, p-toluene sulfonyl chloride (2.0 g, 10.49 mmol) was added, and the reaction mixture was stirred for 4 hours. After 4 hours, the mixture was dropped into 500 mL of acetone, and a white precipitate formed. Then the precipitate was suction-filtered and dried overnight in a vacuum oven at 60 °C. The proton nuclear magnetic resonance (^{1}H NMR) spectrum of the product was detected on a Varian UNITY-plus 400-MHz spectrometer using DMSO-*d6* as a solvent.

1.2 Synthesis of mono-amino-*α*-cyclodextrin (NH_2-*α*CD)

NH_2-*α*CD was synthesized by converting the tosyl group of Ts-α-CD into an amino group through nucleophilic substitution. Briefly, Ts-*α*CD (5.0 g, 4.44 mmol) was dissolved in *N, N*-dimethylformamide at low temperature. Then, potassium carbonate (1.23 g, 8.88 mol) was added, immediately followed by the addition of phthalimide (1.31 g, 8.88 mmol). The mixture was then allowed to warm to room temperature and stirred for 24 hours. The reaction was monitored by thin-layer chromatography (TLC). Then, the solvents were removed under reduced pressure to yield a white solid product, which was dissolved in 100 mL of ethanol, followed by the addition of hydrazine hydrate (80%, 23 mmol). The reaction mixture was gradually heated to reflux for 2 hours. Afterwards, the ethanol was evaporated at reduced pressure. The residue was thoroughly washed with diethyl ether and dried under vacuum to afford the pure NH_2-αCD as a white solid. The molecular weight was determined by high-resolution mass spectromtery (HRMS). MS spectra were recorded on a Varian 7.0T FTMS. All MS experiments were performed using electrospray ionization in positive ion mode.

2. Supporting Figures

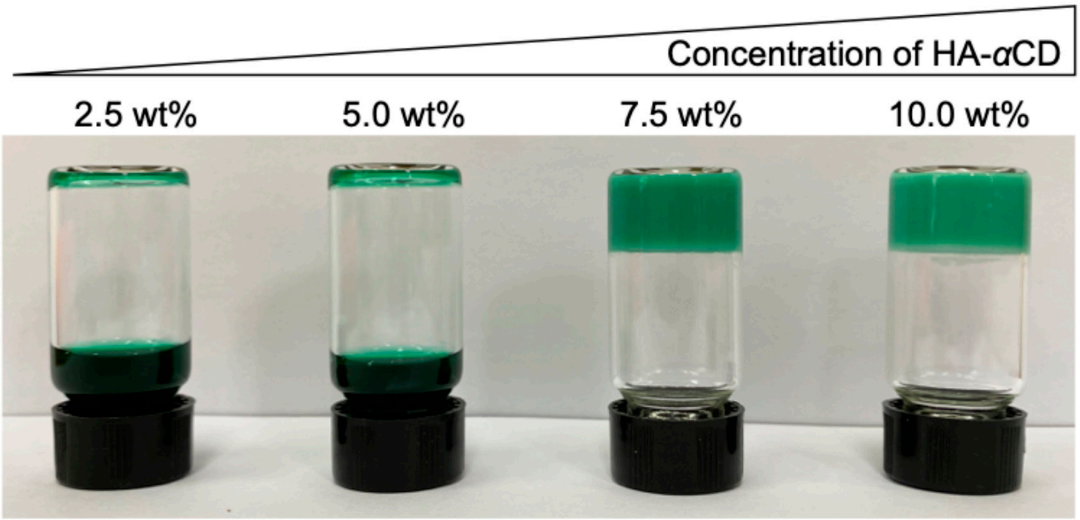

Figure S1. Visual observation of the gelation behavior of IA@PFHA with different HA-αCD concentrations (2.5, 5.0, 7.5, and 10.0 wt%) using the vial inversion method.

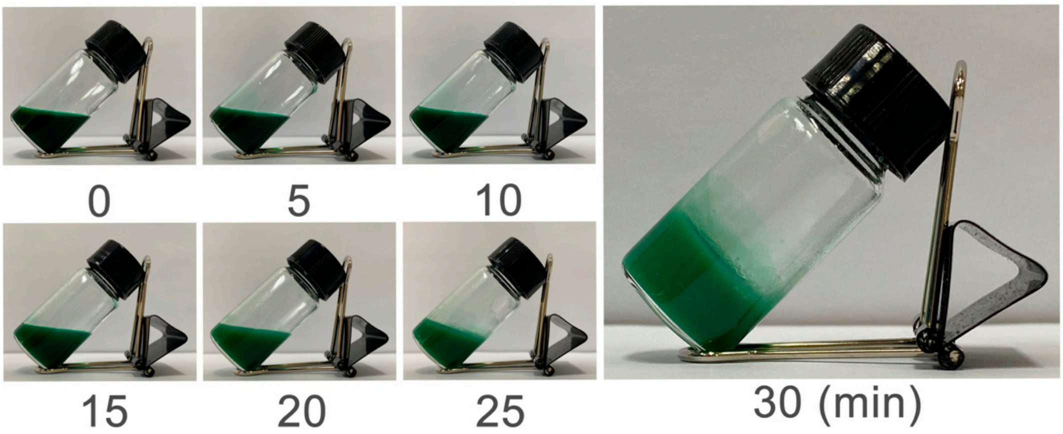

Figure S2. Visual observation of the gelation process of IA@PFHA at room temperature. The gelation time was recorded when the solution ceased to flow.
